# Inorganic Contaminants in Plant-Based Yogurts Commercialized in Brazil

**DOI:** 10.3390/ijerph20043707

**Published:** 2023-02-19

**Authors:** Ana Paula Rebellato, Maria Isabel Andrekowisk Fioravanti, Raquel Fernanda Milani, Marcelo Antonio Morgano

**Affiliations:** 1 Institute of Food Technology, São Paulo 13070-178, SP, Brazil; 2 Adolfo Lutz Institute, São Paulo 01246-902, SP, Brazil

**Keywords:** plant-based, inorganic contaminants, estimated dietary intake, food safety, public health, ICP-MS

## Abstract

This study aimed to evaluate the content of 11 inorganic elements (Al, Cr, Co, Ni, As, Mo, Cd, Sb, Ba, Hg, and Pb) in commercial plant-based and animal-based yogurts for comparison purposes. The samples were mineralized using a simple and fast ultrasound-assisted acid digestion method at 80 °C for 35 min, and the determination of inorganic elements was performed by ICP-MS. The method was validated according to the INMETRO guide, obtaining recoveries from 80 to 110%, precision from 6 to 15%, and a limit of quantification (LOQ) ranging from 200 µg/kg (Al) to 4 µg/kg (other elements). The element concentrations in the plant-based yogurts were Al(<LOQ-9019.05); Cr(<LOQ-88.14); Co(<LOQ-40.56); Ni(31.71-700.46); As(<LOQ-10.61); Mo(<LOQ-355.70); Cd(<LOQ-4.37); Sb and Hg(<LOQ); Ba(<LOQ-1505.71), and Pb(<LOQ-21.58) µg/kg. The elements Mo and Ba were quantified only in the animal-based yogurts, with levels of 72.54 and 160.76 µg/kg, respectively. The results showed a large variation in the concentration of inorganic elements, which demonstrates the importance of knowing the composition of plant-based foods to ensure the safety and health of consumers.

## 1. Introduction

The plant-based food market is growing rapidly, and consumers are increasingly looking for plant-based alternatives to animal products. Several factors, including sustainability, ethics, animal protection, health concerns such as lactose intolerance, cow milk allergy, and heart disease caused by high cholesterol levels, as well as the growth in vegetarian and vegan diets, have contributed to the increased demand for these products, which are low in fat and cholesterol [1,2]. Given the impact of this demand, the food industry has invested in the development of new products, including non-dairy products, based on a variety of plant sources, including seeds, nuts, legumes, cereals, pseudocereals, and others [1].

Yogurt is a popular food with a distinctive flavor and aroma. It is one of the most consumed dairy products in the world, and it is originally produced from the fermentation of cow’s milk by the action of lactic acid bacteria [3]. It is known that its regular consumption helps regulate intestinal transit, it is more easily digested than milk, it helps regulate the immune system, and it contributes to the recommended nutritional needs [4]. However, the demand for alternative plant-based foods as substitutes for dairy products has also caught the attention of consumers, with an emphasis on plant-based yogurts [5,6].

Despite the healthy appeal and the increased consumption of plant-based products, there is a variation in nutritional composition; thus, studies on vegan yogurts are required to use them as a substitute for animal-based yogurts [7]. The nutritional properties of this type of product may vary depending on the raw material, (oilseeds, legumes, cereals, fruits, rice, among others), the fortifiers used, and also the type of manufacturing process [8]. The composition of the raw material can contribute to the presence of bioactive compounds, fibers, vitamins, and minerals in the final product, with positive impacts on human health [2,7,9]. However, toxic elements can also be present in the final product, along with the essential elements. The safety of plant-based foods depends not only on the characteristics of the raw materials used, but also on the cultivation soil, agricultural inputs used in the source plants, harvesting, storage, transport, processing, and post-processing handling. Thus, monitoring the levels of inorganic elements considered toxic is necessary for this type of food [7,8,10].

The toxic mechanisms of inorganic elements include cumulative toxicity and non-carcinogenic and/or carcinogenic risk, with harmful impacts on human health [11]. Inorganic elements such as aluminum (Al), chromium (Cr), cobalt (Co), nickel (Ni), arsenic (As), cadmium (Cd), antimony (Sb), mercury (Hg), and lead (Pb), among others, are considered toxic metals because they cause damage even at low exposure levels. These heavy metals cause damage to various organs after leaving the systemic circulation. However, the great problem associated with these elements is their accumulation over time in various organs and the damage generated by mechanisms such as the breakdown of enzymes, hormones, proteins, and cell membranes, among others, as they have no biological function [12,13,14,15,16,17,18]. There are also reports of long-term human exposure to low levels of barium (Ba), which is associated with symptoms of diarrhea, cardiac arrhythmia, muscle weakness, anxiety, and nervous system disorders [19,20]; thus, it requires evaluation since its presence is widespread in the environment [20]. Despite the few studies, molybdenum (Mo) has been investigated due to the risk of cardiovascular diseases and diabetes in the population, mainly by exposure due to diet habits [21].

Due to the toxicity and associated risks, international bodies such as the JECFA (Joint FAO/WHO Expert Committee on Food Additives), EFSA (European Food Safety Authority), FDA (Food and Drug Administration), and Brazilian bodies such as ANVISA (National Health Surveillance Agency) and MAPA—DIPOV (Ministry of Agriculture, Livestock, and Supply—Department of Inspection of Products of Plant Origin) have defined the maximum limits for inorganic contaminants for several food categories, in addition to safety considerations and risk assessment of chemicals in food [22,23,24,25].

In Brazil, Resolution 722/22 laid out the tolerable maximum limits (TML) of contaminants in food, the general principles for the establishment, and the methods of analysis for conformity assessment [26]. In addition, the Normative Instruction IN 160/22 also established the tolerable maximum limits (TML) of contaminants in food [27]. However, to date, there is still no regulation establishing the maximum limits for inorganic contaminants in plant-based foods. Thus, to evaluate the quality and ensure the food safety of plant-based products for the population, the present study investigated the concentration of 11 potentially toxic inorganic elements by inductively coupled plasma mass spectrometry in 43 samples of plant-based yogurts, and the results were compared with those of yogurt of animal origin.

## 2. Materials and Methods

### 2.1. Reagents and Solutions

For the analytical procedures, the following analytical-grade reagents were used: water purified in a reverse osmosis system with a resistivity less than 18.2 MΩ cm (Gehaka, São Paulo, Brazil); concentrated nitric acid purified by sub-boiling distillation (Distillacid, Berghof, Eningen, Germany); and 30% hydrogen peroxide (H_2_O_2_) (*v*/*v*) (Merck, Darmstadt, Germany). Certified reference material (CRM): INCT-TL-1 Tea leaves (Institute of Nuclear Chemistry and Technology, Warszawa, Poland) and ERM-BD 151 skimmed milk powder. Standard solutions: multi-element (100 mg/L) (Al, Cr, Co, Ni, As, Mo, Cd, Ba, and Pb), Sb (1000 mg/L), and Hg (100 mg/L), all from Specsol (Quimlab, Jacareí, Brazil).

### 2.2. Equipment

The concentrations of the 11 inorganic elements were determined by inductively coupled plasma mass spectrometry ICP-MS (iCAP RQ, Thermo Scientific, Bremen, Germany) and the equipment conditions were set according to the manufacturer’s guidelines, as described in Table 1. An ultrasonic bath (Easy 180H, Elma, Germany) was used for the acid digestion of the samples.

### 2.3. Sampling

From August to October 2022, 43 samples of plant-based yogurt and a sample of natural yogurt of animal origin were purchased from commercial establishments in the municipality of Campinas (São Paulo, Brazil), totaling 5 brands and 17 different flavors. For each sample, 1 to 3 distinct lots were purchased, as shown in Table 2.

### 2.4. Analytical Control

Certified reference materials (INCT-TL-1 Tea leaves (Institute of Nuclear Chemistry and Technology, Warszawa, Poland, ERM-DB 151), skimmed milk powder (Joint Research Center, Geel, Belgium), Tort-3 Lobster Hepatopancreas (National Research Council, Ottawa, Canada), and a plant-based yogurt sample (Sample F, Brand IVV, Lot 1) were used for method validation concerning the figures of merit: accuracy, precision, linearity, the limit of detection, and limit of quantification [28,29].

The accuracy was evaluated using certified reference material (CRM): tea leaves for Al, Ni, Cd, Sb, Ba, and Pb; skimmed milk powder for Mo, Hg, and Pb; and Lobster Hepatopancreas for Co and As. Precision was performed by calculating the coefficient of variation of 7 independent repetitions of the yogurt sample. The limits of detection and quantification were estimated from the analyte concentration corresponding to the average of a blank sample plus three and five standard deviations, respectively.

Certified standard solutions were used to construct the analytical curve: multi-elemental (100 mg/L), Sb (1000 mg/L), and Hg (100 mg/L). The analytical curves were prepared from dilutions of the standard solutions, with five points ranging from 0.1 to 100 μg/L for Cr, Co, Ni, As, Mo, Cd, Sb, Ba, Hg, and Pb, and 5 to 100 µg/L for Al. The standard solutions of Sc, Ge, In, Rh, Bi, and Pt at 1000 mg/L (Fluka, Steinheim, Germany) were used as an internal standard solution at the concentration of 50 µg/L to correct for matrix and instrument deviations.

### 2.5. Sample Preparation

The samples were subjected to ultrasound-assisted acid digestion, as described by Fioravanti et al. (2020) [30], with modifications. Approximately 0.5 g of sample was weighed into a graduated tube (50 mL), 4 mL of HNO_3_ and 2 mL of H_2_O_2_ were added, and the closed tube was kept overnight for approximately 17 h. Then, the tubes were heated in an ultrasonic bath at 80 °C for 35 min. At the end of the mineralization, the digest was cooled down to room temperature, and the volume was made up to 20 mL with ultrapure water and filtered with a 0.45 µm PTFE filter (Agilent Technologies, Tokyo, Japan). All mineralization procedures were performed in triplicate, including the analytical blank.

### 2.6. Inorganic Element Exposure Assessment

The estimated dietary intake of inorganic elements was determined for individuals weighing 15 kg (child) and 60 kg (adult), as well as the levels of occurrence and the consumption of one unit per day. The packages of the plant-based yogurts contained different amounts of samples (90 to 250 g). The amount of the package was taken into account to calculate the exposure, and the results were expressed as micrograms of metal per kg body weight.

The estimated dietary intake was calculated using the deterministic model [31], with the maximum content of the element in the samples analyzed.

To assess the risk associated with exposure to inorganic elements through the consumption of plant-based yogurts, the estimated intake values were compared to the available PTWI (Provisional Tolerable Weekly Intake), PTMI (Provisional Tolerable Monthly Intake), or BMDL (Benchmark Dose Lower Limit), as established by the Joint FAO/WHO Expert Committee on Food Additives (JECFA) and EFSA Panel on Contaminants in the Food Chain (CONTAM Panel).

### 2.7. Statistical Analysis

The results were expressed as the mean and standard deviation ($\bar{x}$ ± SD) of three independent analytical replicates. Analysis of variance (one-way ANOVA) was used to analyze the results, and Tukey’s test (*p* < 0.05) was used for the comparison of means, using the software Statistic 7.0 (StatSoft, Tulsa, OK, USA).

The multivariate analysis was conducted by Principal Component Analysis (PCA) using the software Piroutte 3.11 (Infometrix, Inc., Bothell, WA, USA).

## 3. Results and Discussion

### 3.1. Analytical Method Validation for Al, Cr, Co, Ni, As, Mo, Cd, Sb, Ba, Hg, and Pb in Plant-Based and Animal-Based Yogurts

Linearity was evaluated using five-point analytical curves for each element studied. The analytical curves were linear, with r^2^ > 0.99 for all elements. The limit of detection (LOD) was 119 µg/kg for Al and 2 µg/kg for the other elements (Cr, Co, Ni, As, Mo, Cd, Sb, Ba, Hg, and Pb), and the limit of quantification (LOQ) was 200 µg/kg for Al and 4 µg/kg for the other elements. The tendency/recovery was performed with certified reference material, and the percent recovery ranged from 80 to 110%; the precision was performed on seven repetitions per sample, ranging from 6 to 15%, which met the CV specifications provided by INMETRO [28].

### 3.2. Potentially Toxic Inorganic Elements in Plant-Based and Animal-Based Yogurts

The concentration of the 11 inorganic elements determined in the plant-based yogurts is presented in Table 3.

Large variations of the elements were observed in the different samples, including samples of the same brand and different lots. The analyses were also performed for the animal-based yogurts for comparison purposes, and all elements showed levels below the limit of quantification, except for Mo and Ba, with values of 72.54 and 160.76 µg/kg. This result may be due to the changes in the absorption and accumulation of inorganic elements in foods of animal and plant origin.

As reported in the literature, the safety of plant-based foods depends not only on the characteristics of the raw materials used, but also on the soil, agricultural inputs, harvesting, storage, transport, processing, and post-processing handling. Thus, monitoring the levels of inorganic elements considered potentially toxic is necessary for this type of food [7,8,10,13].

#### 3.2.1. Aluminum

Concerning the Al levels in the plant-based yogurts, the highest concentration (9019.05 µg/kg) was observed for sample M (brand IBT, lot 3), which contained sugar syrup, vegetable fat, cocoa powder, and soy protein isolate, followed by sample C (IVV, lot) (5844.72 µg/kg), which also contained protein isolate (pea and non-transgenic soy) and coconut milk as the main ingredients.

Samples F and G (both from IVV) and O (IMD) showed results below the LOQ (200 µg/kg) for the lots studied. The other samples showed significant differences (*p* < 0.05) in at least one of the lots from the same brand.

High Al concentrations were reported by other authors in cereals and cereal products (44,016 mg/kg), and vegetables and vegetable products (4476 mg/kg) [32]. Filippini et al. (2019) [33] found high Al levels (µg/kg) in cereals and their products (2470.30), sweets, chocolates, and cakes (4387.24), vegetables (7370.23), and fresh fruits (353.20).

Antoine et al. [34] evaluated 13 food cultivars (fruits and vegetables) for the potential health risks associated with Al, As, Cd, and Pb levels in Jamaica. The authors reported Al concentrations ranging from 2.58 mg/kg (pumpkin) to 93.12 mg/kg (banana), while the Al level in coconut was 3.28 mg/kg.

The Joint FAO/WHO Expert Committee on Food Additives (JECFA) established that the value of the PTWI (Provisional Tolerable Weekly Intake) for Al should be 2 mg/kg body weight (bw) for all the Al compounds in foods, including food additives [35].

Considering the sample with the highest Al concentration, the dietary intake values for children and adults were 54.1 and 13.5 µg/kg bw, respectively. The maximum estimated exposure values were 2.7% for children and 0.7% for adults when compared to the available PTWI.

Antoine et al. [34] reported estimated dietary intake values for Al of 46.16, 1.39, and 10 (μg/kg bw/day) for banana, cassava, and coconut, respectively.

#### 3.2.2. Chromium

The highest chromium levels were detected in samples D (IVV, lot 3, 88.14 µg/kg), followed by O (IMD, lot 1, 55.52 µg/kg). Although these samples belong to different brands, they contain the same ingredients, including water, coconut cream, organic sugar, modified starch, and additives, water, coconut milk, cassava starch, and additives.

Samples A, B, C, G, H, I, and K showed results below the LOQ in at least one of the lots, and only samples F and J did not differ significantly (*p* > 0.05) among the lots analyzed.

Concerning the sample with the highest Cr level, the estimated dietary intake was 0.99 and 0.25 µg/kg bw for children and adults, respectively.

It has been reported in the literature that the dietary intake considered safe and adequate for Cr is 50 to 200 μg. However, most diets contain less than 60% of the suggested minimum intake of 50 μg. Furthermore, there are no documented reports of Cr toxicity in nutritional studies at levels up to 1 mg/day [36,37]. As observed in the present study, the estimated dietary intake values for Cr were considered low when compared to the dietary food intake considered safe for humans.

#### 3.2.3. Cobalt

The Co levels of the vegan yogurts ranged from <LOQ to 40.56 µg/kg. Sample M (IBT, lots 1 to 3) showed the highest Co concentrations, probably due to the ingredients of the formulation, such as sugar syrup, vegetable fat, cocoa powder, and soy protein isolate.

Five samples showed results below the LOQ (E, F, G, J, and K) in three lots, while only one sample (L) showed no significant difference between the lots studied.

The average dietary intake of Co from food is estimated to be between 5 and 60 μg/day, varying among countries. In France, the average dietary intake of Co is 4 to 29 μg/day; in Canada and the UK, the value is 11 μg/day; and in Turkey, the average value ranges from 60 to 65 μg/day [38].

Although the dietary intake of Co is low, food is the major source of exposure for the general population. Therefore, the estimated dietary intake of 1 unit of plant-based yogurt with a higher Co level was determined, with values of 0.24 and 0.06 ug/kg bw for children and adults, respectively.

Food sources rich in cobalt include meat muscle, liver, fish, nuts, oats, and green leafy vegetables (broccoli and spinach). Studies have reported average concentrations of 0.25–1.03 μg/g in dried fruits; 0.06–0.18 μg/g in cruciferous vegetables (*Brassicaceae*), 0.02–0.06 μg/g in cereals, and 0.17 μg/g in fish liver, analyzed by ICP-OES or ICP-MS [38]. The results reported by those authors were higher than those observed in this study.

#### 3.2.4. Nickel

The samples Q and P, both from the same brand (IPV), had the highest Ni concentrations (700.46 and 491.08 µg/kg, respectively). These samples were purchased in a pack and in powder form, requiring reconstitution before consumption. In addition, both samples presented coconut milk powder as the main ingredient, with additives in their composition. No significant differences (*p* > 0.05) were observed among samples E, F, G, H, J, L, and M when comparing the different lots.

Cubadda et al. [39] conducted a study on the dietary exposure of the Italian population to Ni by evaluating over 3000 food matrices that tallied 51 food groups present in the Italian diet. The Ni contents ranged from 7355 (cocoa) to <1 μg/kg fresh weight (bottled water).

In 2020, the European Commission, through the EFSA Panel on Contaminants in the Food Chain (CONTAM), updated their opinion on nickel levels in food and drinking water according to the new occurrence data from the benchmark dose (BMD) approach in risk assessment. From the updated BMD analysis, the BMDL_10_ of 1.3 mg Ni/kg body weight per day was selected as the reference for establishing the tolerable dietary intake (TDI), and a TDI of 13 ug/kg body weight was established by applying the standard uncertainty factor of 100, aimed to account for intra- and inter-species differences [40].

Thus, considering the sample with the highest Ni level as a reference, the estimated intakes for children and adults ranged from 6.1 and 1.5 µg/kg bw, respectively. The maximum estimated intake was compared to the TDI of 13 ug/kg bw established by the EFSA [40], with values of 46.7 and 11.7% for children and adults, respectively. This scenario shows that the consumption of this product should done with caution, especially for children, since the consumption of 2 units per day corresponds to 93.4% of the recommended TDI for this element.

#### 3.2.5. Arsenic

Arsenic levels were detected only in samples L (IFR, lot 3), M (IBT, lot 1), and P (IPV), with values of 10.61, 4.33, and 4.38 µg/kg, respectively. The remaining samples had As concentrations below the LOQ.

In 2021, EFSA published a study on chronic dietary exposure to inorganic arsenic, with the evaluation of different food matrices. The mean lower and upper levels ranged from 0.007–4 (cow milk) to 9113–9134 (kombu) µg/kg [41]. High As concentrations have also been observed in aquatic products and their derivatives (0.728 mg/kg). In addition, mean levels of 0.118 mg/kg have been reported in vegetables and plant-derived products [32].

Although there are different international standards or regulatory limits for the presence of arsenic in food and drinking water, usually those reported by the EFSA—Panel on Contaminants in the Food Chain (CONTAM Panel) and the Joint FAO/WHO—Expert Committee on Food Additives (JECFA) are usually well accepted. In 2009, the EFSA established a reference value between 0.3 and 8 µg/kg bw per day as the benchmark lower confidence limit (BMDL_0.1_) [42]. In 2011, the JECFA identified a BMDL_0.5_ of 3.0 µg/kg bw per day for an increased risk of lung cancer [43].

Based on the value established by the JECFA, the maximum dietary intake was calculated for sample L, with values of 0.08 and 0.02 µg/kg bw for children and adults, respectively. The maximum estimated dietary intake was compared with the BMDL_0.5_ of 3.0 µg/kg bw per day, established by the JECFA [43], with values of 2.6 and 0.6% for children and adults, respectively.

Dietary exposure values of As of 0.30 ug/kg bw per day for infants, 0.61 ug/kg bw per day for children, and 0.03 to 0.15 ug/kg bw per day for the adult population (adults, elderly, and very elderly) were estimated (EFSA, 2021), which were higher than those observed for the samples of the present study.

#### 3.2.6. Molybdenum

The highest Mo contents were observed for samples A, B, C (IVV brand, lot 1 to 3), and M (IBT, lot 1 to 3), which ranged from 190.39 to 355.70 µg/kg. The higher Mo concentration in these yogurts may be due to their similar composition. Samples A, B, and C were from the same brand and had protein isolates (pea and non-transgenic soy) as the main ingredient, and sample M also contained soy protein isolate. The other samples did not have these ingredients in their composition, and the Mo contents varied from <LOQ to 37.83 µg/kg.

No significant differences (*p* > 0.05) were observed for the molybdenum levels of sample M when compared to all lots studied. Moreover, the yogurt of animal origin (R) showed a higher Mo level (72.54 µg/kg) than the samples that did not contain plant proteins, and it had a lower level than the plant-based samples.

The estimated dietary intake of the plant-based yogurts with higher Mo concentrations was determined, and values of 5.93 and 1.48 µg/kg bw were observed for children and adults, respectively.

In 2013, the European Food Safety Authority (EFSA) published their Scientific Opinion on Food Reference Values for Molybdenum, stating that there is insufficient evidence to derive a Mean Requirement and a Population Reference Intake (PRI) for this element. Therefore, an adequate intake (AI) of 65 µg/day for adolescents and adults, 20 µg/day for children aged 4 to 6 years, and 10 µg of Mo per day for children aged 7 to 11 months have been proposed [44].

The adequate intake value was determined for sample B, with a value of 88.9 µg/day. Taking into account the EFSA recommendation for AI, this result was higher for both adult and child consumption. Thus, moderate consumption of this product is recommended, since other foods containing Mo can also be consumed during meals.

Molybdenum exposure occurs mainly through food consumption. Studies have reported an average dietary intake of 109 and 76 mg/day for men and women, respectively, in the United States. In European countries, the average intake ranged from 58 to 157 µg/day [21]. Also, average intakes of 87, 94, 157, and 124 µg/day have been reported in Belgium, France, Sweden, and the United Kingdom, respectively [21]. Foods that contribute the most to dietary Mo intake include cereal products (50%), dairy products (11% to 16%), and vegetables (10 to 20%) [21].

#### 3.2.7. Cadmium

The element cadmium was detected in only two samples (M, IBT, lot 2, and Q, IPV) of the 44 samples studied, with values of 4.20 and 4.37 µg/kg, respectively, while concentrations below the LOQ were observed for the other samples.

Wang et al. assessed the Pb, As, Cd and Al content in 12 food categories, including cereals and cereal products; beans, nuts, and their products; potatoes and their products; milk and dairy products; vegetables and vegetable products; and fruits and fruit products. The authors observed mean Cd levels of 0.0240 mg/kg in beans, nuts, and their derivatives, especially the peanuts (0.0841 mg/kg); 0.011 mg/kg for vegetables and derived products; and 0.004 mg/kg for fruits and derived products [32]. Cd was also found in fruits and vegetables in a study in Jamaica, with levels ranging from 0.286 (turnip) to 0.015 (pumpkin) mg/kg, and 0.079 mg/kg in coconut samples [34].

The Joint FAO/WHO Expert Committee on Food Additives has adopted a provisional tolerable monthly intake (PTMI) for Cd of 25 µg/kg bw per month, corresponding to a tolerable weekly intake (TWI) of approximately 6 µg /kg bw per week [45]. In turn, the European Food Safety Authority has established a tolerable lower weekly intake of 2.5 µg/kg bw to ensure a high level of protection for all consumers, especially vulnerable subgroups [46].

The maximum dietary intake values for Cd were calculated for the plant-based yogurt, with values of 0.032 and 0.008 µg/kg bw for children and adults, respectively. When compared to PTMI, estimated maximum exposure values were 3.9 and 1.0% for children and adults, respectively.

Some reports on the average dietary cadmium exposure ranged from 0.6 μg/kg bw per month (2.4% of PTMI) for adults in the Sikasso region (Mali) to 24 μg/kg bw per month (96% of PTMI) in children aged 4 to 11 years in China [41]. Antoine et al. [34] reported the estimated dietary intake of Cd for coconut, tomatoes, carrots, and bananas, with values of 0.240, 0.116, 0.137, and 0.028 ug/kg bw/day, respectively, which were higher than those observed in the present study.

#### 3.2.8. Antimony and Mercury

All samples showed results below the LOQ for the elements Sb and Hg.

For mercury, the Joint FAO/WHO Expert Committee on Food Additives (JECFA) established a PTWI of 1.6 μg/kg bw per week, which was adopted in the European Union and Norway. The US EPA has established a reference dose (RfD) of 0.1 μg/kg bw per day [43,47]. From a dietary standpoint, mercury exposure occurs mainly through the consumption of fish and seafood, which contain 10 to 100 times higher levels when compared to other foods such as cereals, potatoes, vegetables, fruits, meat, poultry, eggs, milk, and dairy products [48].

Regarding Sb, although it was not detected in the samples of this study, levels of 1 to 10 ng/g wet weight have been reported in meat, freshwater fish, poultry, cereals, fruits, and vegetables, as well as in human milk (13 μg/kg) and ready-to-eat products (0.22 to 2.81 μg/kg) [49].

#### 3.2.9. Barium

The Ba levels of the samples of this study ranged from <LOQ to 1505.71 µg/kg. The highest contents were found in sample L (IFR brand, lots 1 to 3, 1072.76–1505.71 µg/kg), followed by sample M (IBT, lots 1 to 3, 527.17–580.98 µg/kg). The difference between these samples when compared to the other samples is the presence of red fruits (strawberry, blackberry, and blueberry), demerara sugar, chia seeds, sugar syrup, vegetable fat, and cocoa powder, respectively, in the formulations.

No significant differences (*p* > 0.05) in Ba concentration were observed for four sample (B, H, J, and M) when compared to all lots studied, while the yogurt of animal origin showed a Ba level of 160.76 µg/kg.

In the literature, reports have shown relatively low (<0.1 mg/kg) barium levels in foods of animal origin (milk, meat, and fish), and higher levels in plant-based foods (around 0.5 mg/kg), while cereal products have concentrations of around 1 mg/kg. Higher Ba levels have been reported in nuts and other chestnuts (131–3000 mg/kg), chewing gum (5 mg/kg), and herbs and spices (34 mg/kg) [50].

The World Health Organization (WHO) has established a value of 20 µg Ba/kg bw per day as adequate for the characterization of risk assessment [50,51]. The maximum dietary intake was determined for the plant-based yogurts, with values of 11.03 and 2.76 µg Ba/kg bw for children and adults, respectively.

The dietary intake of barium has been estimated in some studies, ranging from 6 to 10 μg/kg bw per day in adults [50,52]. Higher exposure was seen in infants and children, with average barium intakes of 10 and 27 mg/kg bw per/day, respectively. Nuts are considered the main sources of dietary exposure, followed by bread and other cereal products, vegetables, and fruits [50].

#### 3.2.10. Lead

Most samples showed Pb levels lower than the LOQ, while samples D, E, J, M, and P exhibited values ranging from 4.39 to 21.58 µg/kg. In addition, sample M presented the highest contents in the two lots evaluated when compared to the other lots, probably due to the ingredients in its composition (sugar syrup, vegetable fat, and cocoa powder).

Mean Pb concentrations (µg/kg) were reported by Wang et al. [32] in eight cereal-based foods; forty-five samples of beans, nuts, and derivatives; seven dairy products; twelve plant-based foods; six fruits and derivatives; and three samples of water and beverages. Mielech et al. [53] conducted a survey on the risk of contamination of baby and infant foods, including Pb. A study showed that 96% of infant formulas exceeded the daily allowable Pb concentration (0.4 mg/kg), while another study reported that 37% of infant samples had Pb contamination. In addition, higher Pb levels were reported in rice-based products [53].

Although FAO/WHO withdrew the PTWI of 25 μg/kg bw per week for Pb, the EFSA, through the Panel on Contaminants in the Food Chain (CONTAM Panel), identified neurotoxicity in children and cardiovascular effects and nephrotoxicity in adults as critical risk assessment effects. Therefore, a dietary intake value of 12 µg/kg bw per day was established (BMDL_0.1_) [45,54].

Daily tolerable limits of Pb were reported for children and adults, with values of 0.13 and 0.03 µg/kg bw, respectively, and estimated maximum exposures of 1.08 and 0.27%, respectively, when compared to BMDL.

In a Scientific Opinion on Lead in Foods, the EFSA reported that lead dietary exposure in adults ranged from 0.36–1.24 to 2.43 µg/kg bw per day for medium and high consumption in Europe, respectively. Further, exposure in infants was reported to range from 0.21 to 0.94, and in children from 0.80 to 3.10 (moderate consumer) and up to 5.51 (high consumer) µg/kg bw per day. In addition, cereal products contributed most to lead dietary exposure, whereas dust and soil may be important non-food sources of contamination in children [54]. Estimated dietary intake values for Pb of 0.002 to 0.064 µg/kg bw per day for fruits and vegetables have also been reported by Antoine et al. [34].

### 3.3. Multivariate Analysis

**Figure 1:**
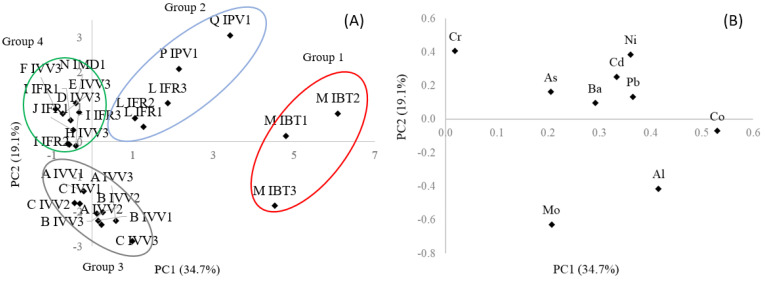
Principal Component Analysis of toxic inorganic elements in plant-based yogurts. Graphs of scores (**A**) and loadings (**B**). Group 1 (red circle): sample of brand M; Group 2 (blue circle): samples of brands L, P and Q; Group 3 (gray circle): samples of brands A, B and C; Group 4 (green circle): samples of brands D, E, F, G, H, I, J, K, N, O and R.

Principal Component Analysis (PCA) was performed as an exploratory tool to evaluate the results and to determine possible correlations between the samples and the variables studied. The PCA was performed using a matrix (44 × 9), wherein the lines and columns represent the forty-four samples and the nine variables (inorganic elements Al, Cr, Co, Bi, As, Mo, Cd, Ba, and Pb), respectively. Sb and Hg were not included since they were found at non-detected levels in plant-based beverages, and data were scaled to provide the same weight in the model. The PCA output was the scores (samples) and loading (inorganic elements) plots, presented in Figure 1.

The score graph (Figure 1A) shows the sample distribution with the formation of four distinct groups, and the loadings (inorganic elements) graph (Figure 1B) indicates the variables and the effects that led to group separation.

The score graph shows the sample distribution, classifying the samples in four distinct groups. The first group is formed by samples M (Naturis soy chocolate flavor—same brand and different lots) which presented higher Co and Al levels. The second group is composed by samples L (Iog Veg berry fermented coconut cream), P (red fruit coconut yogurt) and Q (coconut yogurt), which contained Ni, Ba, Cd and As in their composition. The third group is composed by samples A, B, and C (same brand, pea and soy protein as main ingredient and declared as “veg protein”), which presented the highest Mo level. The fourth group is composed of the remaining samples, including sample R (yogurt of animal origin), probably classified due to their low concentrations of all elements, except for Cr.

## 4. Conclusions

A simple and rapid sample preparation method using ultrasound-assisted digestion and ICP-MS was studied for the evaluation of 11 inorganic elements considered potentially toxic in different plant-based yogurts. The method showed adequate linearity, the limit of detection, and the limit of quantification, precision, and recovery for the quantification of the inorganic elements under study.

Concerning the elements evaluated in the plant-based samples, a large variation was observed in the concentration of inorganic elements, even for different lots of the same brand. When comparing the results with the animal-based yogurt, most of them showed levels lower than the LOQ, except for Mo and Ba. The estimated dietary intake for children and adults was determined for the inorganic elements under study and compared with the health-based guideline for PTWI, PTMI, and BDML, when available. For the element Mo, the calculated adequate intake was higher than the recommended value; thus, special attention is needed on the amount of plant-based yogurt consumed that does not exceed the recommended intake.

The exploratory analysis (PCA) allowed the separation of the samples into four groups with distinct characteristics of composition, both concerning the ingredients and the concerning inorganic elements evaluated, although most samples contained coconut as the basis of the composition.

The major problem associated with the presence of these inorganic elements in foods is their cumulative toxicity, non-carcinogenic risk, and carcinogenic risk, causing harmful impacts on human health.

Thus, extensive knowledge about the composition of inorganic elements in novel foods is required to ensure the safety and health of consumers, mainly regarding the plant-based category. The results of this study can contribute to the establishment of maximum tolerable limits for inorganic contaminants in plant-based yogurts.

## Figures and Tables

**Table 1:** ICP-MS parameters used for the determination of inorganic contaminants in yogurt samples.

Power (RF)	1550 W
Air flow rate/Auxiliary air	14.0/0.80 L/min
He flow rate	5.00 mL/min
Nebulizer flow rate	Micromist; 0.98 L/min
Dwell time	0.3 s/0.02 s (IS)
Monitored isotopes	^27^Al, ^53^Cr, ^59^Co, ^60^Ni, ^75^As, ^97^Mo, ^111^Cd, ^123^Sb, ^137^Ba, ^202^Hg, ^208^Pb
Internal standard (IS) (50 µg/L)	^45^Sc; ^72^Ge; ^115^In; ^103^Rh; ^209^Bi; ^195^Pt

**Table 2:** Identification and labeling information (description and ingredients) of the plant-based and animal-based yogurt samples.

Sample	Brand	Description	Lot (n)	Ingredients
A	IVV	Veg Protein cookies and cream	3	Water, protein isolates (pea protein and non-GMO soy protein), organic sugar, coconut cream, emulsifier (sunflower lecithin), stabilizer (pectin), nature-identical flavor, preservative (potassium sorbate), sweetener (stevia), vegan yeast
B	IVV	Veg Protein peanut butter	3	Water, protein isolates (pea and non-transgenic soy), coconut milk, organic sugar, peanut preparation (water, organic sugar, peanut-identical flavor, natural dye, acidulant citric acid, stabilizer pectin, preservative potassium sorbate), emulsifier (sunflower lecithin), stabilizer (pectin), nature-identical flavor, preservative (potassium sorbate), sweetener (stevia), vegan yeast
C	IVV	Veg Protein strawberry	3	Water, protein isolates (pea and non-transgenic soy), coconut milk, organic sugar, strawberry preparation (water, organic sugar, strawberry pulp, strawberry-identical flavor, acidulant citric acid, stabilizer pectin, preservative (potassium sorbate), sweetener (stevia), vegan yeast
D	IVV	Iog Veg Coconut (yogurt-flavored coconut food)	3	Water, coconut cream, organic sugar, modified starch, soluble fiber, tricalcium phosphate (calcium), xanthan gum stabilizer, natural coconut flavor, potassium sorbate preservative, and yeast
E	IVV	Iog Veg strawberry	3	Water, coconut cream, organic sugar, strawberry preparation (water, strawberry, maltodextrin, modified starch, natural dyes anthocyanins and annatto, flavoring, xanthan gum thickener, potassium sorbate preservative, and lactic acid acidulant), modified starch, soluble fiber, tricalcium phosphate (calcium), xanthan gum stabilizer, potassium sorbate preservative, and yeast
F	IVV	Iog Veg banana, papaya, and apple	3	Water, coconut cream, organic sugar, banana, papaya, and apple preparation (water, maltodextrin, banana, apple and papaya, modified starch, natural dyes anthocyanins and annatto, flavoring agent, acidulant lactic acid and preservative potassium sorbate), modified starch, soluble fiber, tricalcium phosphate (calcium), stabilizer xanthan gum, preservative potassium sorbate, and yeast
G	IVV	Grego Veg traditional	3	Coconut milk (water and coconut cream), organic sugar, modified starch, stabilizer (pectin), preservative (potassium sorbate), and yeast
H	IVV	Grego Veg strawberry	3	Coconut milk (water and coconut cream), organic sugar, modified starch, strawberry, apple juice, stabilizer (pectin), preservative (potassium sorbate), natural coloring (beet), and yeast
I	IFR	Iog Veg apricot fermented coconut cream	3	Water, apricot, coconut pulp, potato starch, agar agar, natural sweetener steviol glycosides, preservative potassium sorbate, and fermentation cultures (*Streptococcus salivarius* ssp. *thermophillus* and *Bifidobacterium animalis* ssp. *lactis*)
J	IFR	Iog Veg cranberry hibiscus fermented coconut cream	3	Water, cranberry, coconut pulp, potato starch, hibiscus, agar agar, natural sweetener steviol glycosides, preservative potassium sorbate, and fermentation cultures (*Streptococcus salivarius* ssp. *thermophillus* and *Bifidobacterium animalis* ssp. *lactis*)
K	IFR	Iog Veg natural fermented coconut cream	3	Water, coconut pulp, potato starch, inulin, agar agar, potassium sorbate preservative, and fermentation cultures (*Streptococcus salivarius* ssp. *thermophillus* and *Bifidobacterium animalis* ssp. *lactis*)
L	IFR	Iog Veg berry fermented coconut cream	3	Coconut milk (water and coconut pulp), red fruits (strawberry, blackberry, and blueberry), demerara sugar, potato starch, chia, agar agar, fruit pectin, preservative potassium sorbate, and fermentation cultures (*Streptococcus salivarius* ssp. *thermophillus* and *Bifidobacterium animalis* ssp. *lactis*)
M	IBT	Naturis soy chocolate flavor	3	Water, sugar syrup, maltodextrin, vegetable fat, modified starch, cocoa powder, soy protein isolate, sugar, calcium (tricalcium phosphate), salt (sodium chloride), thickener guar gum and carrageenan, flavoring agent, stabilizer tetrasodium pyrophosphate, caramel IV colorings, and acid regulator disodium phosphate
N	IMD	Coconut Cream with Pineapple and Cinnamon	1	Water, coconut milk, pineapple, cassava starch, erythritol, cinnamon, lemon, and potassium sorbate preservative
O	IMD	Natural coconut cream	1	Water, coconut milk, cassava starch, erythritol sweetener, and preservative potassium sorbate
P	IPV	Red fruit coconut yogurt	1	Coconut milk powder, partially hydrolyzed guar (sun fiber), guar and xanthan gums, blackberry, raspberry, strawberry, lactic acid, erythritol, thaumatin, steviol glycoside, natural berry and vanilla flavor, and food grade silica (anti-humectant)
Q	IPV	Coconut yogurt	1	Coconut milk powder, partially hydrolyzed guar (sun fiber), guar and xanthan gums, isomaltulose (palatinose), lactic acid, and natural vanilla flavor
R	INB	Natural yogurt	1	Whole milk and/or reconstituted whole milk, skim milk powder, and lactic acid starter

**Table 3:** Concentration (µg/kg) of 11 potentially toxic inorganic elements in yogurt samples of plant and animal origin.

Samples	Brand	Lot	Al	Cr	Co	Ni	As	Mo	Cd	Sb	Ba	Hg	Pb
A	IVV	1	n.d.	n.d.	5.99 ± 0.48b	76.03 ± 10.58a	n.d.	317.65 ± 17.86b	n.d.	n.d.	250.45 ± 14.43a	n.d.	n.d.
		2	2836.90 ± 97.62b	n.d.	6.08 ± 0.11b	32.68 ± 0.61b	n.d.	348.39 ± 8.47a	n.d.	n.d.	101.77 ± 4.94c	n.d.	n.d.
		3	3097.16 ± 5.66a	4.21 ± 0.94a	10.54 ± 0.04a	40.25 ± 2.09b	n.d.	331.18 ± 2.61ab	n.d.	n.d.	137.67 ± 5.63b	n.d.	n.d.
B	IVV	1	2154.28 ± 82.33b	n.d.	6.80 ± 0.04a	31.55 ± 1.40b	n.d.	355.70 ± 3.66a	n.d.	n.d.	126.90 ± 11.12a	n.d.	n.d.
		2	2746.24 ± 130.29a	9.23 ± 2.48a	4.45 ± 0.13b	35.95 ± 3.16b	n.d.	329.02 ± 18.53ab	n.d.	n.d.	136.52 ± 4.65a	n.d.	n.d.
		3	2803.48 ± 129.80a	5.30 ± 0.52b	6.80 ± 0.35a	42.77 ± 1.34a	n.d.	307.15 ± 9.82b	n.d.	n.d.	144.15 ± 15.25a	n.d.	n.d.
C	IVV	1	2289.29 ± 230.49b	6.51 ± 1.44a	n.d.	39.45 ± 4.67ab	n.d.	293.41 ± 12.01b	n.d.	n.d.	135.14 ± 6.93b	n.d.	n.d.
		2	1580.46 ± 82.71c	n.d.	n.d.	31.71 ± 0.19b	n.d.	294.24 ± 5.55ab	n.d.	n.d.	185.61 ± 7.11a	n.d.	n.d.
		3	5844.72 ± 92.46a	n.d.	7.06 ± 0.57a	47.48 ± 8.44a	n.d.	317.36 ± 9.90a	n.d.	n.d.	145.87 ± 6.93b	n.d.	n.d.
D	IVV	1	n.d.	88.14 ± 1.85a	n.d.	119.12 ± 19.18a	n.d.	10.39 ± 0.58b	n.d.	n.d.	99.29 ± 7.29b	n.d.	n.d.
		2	329.54 ± 12.59b	48.45 ± 8.97b	n.d.	54.57 ± 3.32b	n.d.	n.d.	n.d.	n.d.	155.70 ± 5.70a	n.d.	n.d.
		3	590.99 ± 2.94a	46.91 ± 1.67b	6.90 ± 0.97a	59.01 ± 2.17b	n.d.	14.21 ± 1.72a	n.d.	n.d.	64.52 ± 0.86c	n.d.	4.39 ± 0.61a
E	IVV	1	n.d.	49.10 ± 5.62a	n.d.	59.56 ± 2.19a	n.d.	15.57 ± 1.68a	n.d.	n.d.	51.83 ± 1.53c	n.d.	n.d.
		2	266.83 ± 48.98a	49.01 ± 7.97a	n.d.	55.55 ± 9.10a	n.d.	n.d.	n.d.	n.d.	132.68 ± 9.68b	n.d.	n.d.
		3	n.d.	32.54 ± 1.63b	n.d.	48.39 ± 0.78a	n.d.	12.73 ± 0.91b	n.d.	n.d.	158.82 ± 3.08a	n.d.	5.74 ± 0.32a
F	IVV	1	n.d.	34.7 ± 5.12a	n.d.	49.10 ± 8.42a	n.d.	9.55 ± 1.78a	n.d.	n.d.	n.d.	n.d.	n.d.
		2	n.d.	39.74 ± 2.28a	n.d.	56.37 ± 5.24a	n.d.	n.d.	n.d.	n.d.	47.10 ± 4.06a	n.d.	n.d.
		3	n.d.	42.33 ± 2.76a	n.d.	49.62 ± 1.06a	n.d.	8.76 ± 0.43a	n.d.	n.d.	46.22 ± 3.62a	n.d.	4.69 ± 0.41a
G	IVV	1	n.d.	n.d.	n.d.	66.85 ± 3.91a	n.d.	n.d.	n.d.	n.d.	n.d.	n.d.	n.d.
		2	n.d.	8.46 ± 0.61b	n.d.	57.84 ± 3.62a	n.d.	n.d.	n.d.	n.d.	102.66 ± 7.25a	n.d.	n.d.
		3	n.d.	19.96 ± 0.70a	n.d.	59.27 ± 0.85a	n.d.	8.18 ± 0.17a	n.d.	n.d.	55.65 ± 2.05b	n.d.	n.d.
H	IVV	1	n.d.	4.64 ± 1.01b	n.d.	51.87 ± 1.01a	n.d.	4.76 ± 0.22b	n.d.	n.d.	84.51 ± 19.31a	n.d.	n.d.
		2	n.d.	n.d.	n.d.	58.04 ± 3.00a	n.d.	n.d.	n.d.	n.d.	91.96 ± 4.39a	n.d.	n.d.
		3	1068.53 ± 152.08a	7.65 ± 1.39a	6.30 ± 1.18a	68.86 ± 15.77a	n.d.	6.95 ± 0.18a	n.d.	n.d.	90.67 ± 13.71a	n.d.	n.d.
I	IFR	1	n.d.	n.d.	n.d.	250.60 ± 88.20a	n.d.	19.75 ± 0.35a	n.d.	n.d.	257.02 ± 56.02a	n.d.	n.d.
		2	1044.48 ± 9.78b	n.d.	4.58 ± 0.50a	120.74 ± 6.94b	n.d.	14.42 ± 0.34c	n.d.	n.d.	91.83 ± 9.60b	n.d.	n.d.
		3	1537.15 ± 33.32a	4.59 ± 0.72a	4.93 ± 0.50a	116.43 ± 9.45b	n.d.	16.86 ± 1.11b	n.d.	n.d.	115.98 ± 11.30b	n.d.	n.d.
J	IFR	1	477.47 ± 54.10a	7.92 ± 1.02a	n.d.	58.12 ± 12.29a	n.d.	7.04 ± 0.62c	n.d.	n.d.	293.98 ± 17.65a	n.d.	6.07 ± 0.01
		2	360.93 ± 34.57b	5.73± 0.97a	n.d.	48.93 ± 3.31a	n.d.	9.69 ± 0.29b	n.d.	n.d.	304.21 ± 17.87a	n.d.	n.d.
		3	334.11 ± 0.27b	6.67 ± 0.78a	n.d.	54.86 ± 3.06a	n.d.	12.54 ± 0.49a	n.d.	n.d.	288.29 ± 8.30a	n.d.	n.d.
K	IFR	1	236.82 ± 13.68c	n.d.	n.d.	59.02 ± 2.16a	n.d.	7.63 ± 0.74b	n.d.	n.d.	n.d.	n.d.	n.d.
		2	779.34 ± 61.04a	5.06 ± 0.29a	n.d.	46.15 ± 3.47b	n.d.	10.23 ± 0.45a	n.d.	n.d.	36.36 ± 3.11a	n.d.	n.d.
		3	372.42 ± 59.19b	<LOQ	n.d.	59.58 ± 5.33a	n.d.	12.31 ± 1.40a	n.d.	n.d.	39.94 ± 3.49a	n.d.	n.d.
L	IFR	1	1105.91 ± 121.14a	9.61 ± 2.73b	11.95 ± 0.55a	135.91 ± 26.83a	n.d.	31.96 ± 1.99b	n.d.	n.d.	1505.71 ± 80.39a	n.d.	n.d.
		2	820.81 ± 73.00b	22.68 ± 3.89a	12.34 ± 2.47a	152.30 ± 22.94a	n.d.	37.83 ± 0.86a	n.d.	n.d.	1252.41 ± 190.17ab	n.d.	n.d.
		3	819.33 ± 20.20b	4.26 ± 0.58b	10.91 ± 1.21a	109.33 ± 15.83a	10.61 ± 2.49a	35.69 ± 1.94ab	n.d.	n.d.	1072.76 ± 153.90b	n.d.	n.d.
M	IBT	1	4439.07 ± 151.19b	30.20 ± 2.57b	28.60 ± 0.80b	244.58 ± 11.15a	4.33 ± 0.15a	190.39 ± 2.47a	n.d.	n.d.	534.31 ± 8.29a	n.d.	19.85 ± 3.93
		2	4731.97 ± 289.62b	25.89 ± 2.88b	28.03 ± 2.27a	255.85 ± 6.47a	n.d.	207.61 ± 12.98a	4.20 ± 0.18	n.d.	527.17 ± 36.35a	n.d.	21.58 ± 5.39
		3	9019.05 ± 759.89a	39.50 ± 4.27a	40.56 ± 4.01a	236.38 ± 5.87a	n.d.	207.26 ± 9.09a	n.d.	n.d.	580.98 ± 44.26a	n.d.	n.d.
N	IMD	1	305.56 ± 12.36	48.97 ± 2.28	4.56 ± 0.56	119.85 ± 2.02	n.d.	17.09 ± 0.53	n.d.	n.d.	400.48 ± 3.17	n.d.	n.d.
O	IMD	1	n.d.	55.52 ± 1.93	n.d.	113.49 ± 2.16	n.d.	18.52 ± 0.89	n.d.	n.d.	46.55 ± 7.66	n.d.	n.d.
P	IPV	1	1382.27 ± 136.02	30.69 ± 0.90	12.41 ± 4.58	491.08 ± 40.44	4.38 ± 0.24	15.04 ± 3.12	n.d.	n.d.	349.80 ± 8.85	n.d.	4.33 ± 1.08
Q	IPV	1	859.20 ± 56.22	12.57 ± 1.31	14.93 ± 1.93	700.46 ± 23.50	n.d.	30.50 ± 1.37	4.37 ± 0.28	n.d.	190.39 ± 7.67	n.d.	n.d.
R	INB	1	n.d.	n.d.	n.d.	n.d.	n.d.	72.54 ± 1.21	n.d.	n.d.	160.76 ± 5.59	n.d.	n.d.

Results are expressed as mean ± standard deviation (n = 3). Mean with different letters in the same column and in the same sample indicate a significant difference (*p* < 0.05) by one-way ANOVA and Tukey’s test at 95% of confidence; n.d.: not detected (<LOQ), (LOQ = Al: 200 µg/kg; Cr, Co, Ni, As, Mo, Cd, Sb, Ba, Hg, and Pb: 4 µg/kg).

## Data Availability

The data that support the findings of this study are available upon reasonable request.

## References

[1] Mäkinen O.E., Wanhalinna V., Zannini E., Arendt E.K. (2016). Foods for Special Dietary Needs: Non-dairy Plant-based Milk Substitutes and Fermented Dairy-type Products. Crit. Rev. Food Sci. Nutr..

[2] Sethi S., Tyagi S.K., Anurag R.K. (2016). Plant-based milk alternatives an emerging segment of functional beverages: A review. J. Food Sci. Technol..

[3] Lee W.J., Lucey J.A. (2010). Formation and Physical Properties of Yogurt. Asian-Australasian J. Anim. Sci..

[4] Benmeziane F., Raigar R.K., Ayat N.E.-H., Aoufi D., Djermoune-Arkoub L., Chala A. (2021). Lentil (Lens culinaris) flour addition to yogurt: Impact on physicochemical, microbiological and sensory attributes during refrigeration storage and microstructure changes. LWT.

[5] Klost M., Drusch S. (2019). Structure formation and rheological properties of pea protein-based gels. Food Hydrocoll..

[6] Yang X., Su Y., Li L. (2020). Study of soybean gel induced by Lactobacillus plantarum: Protein structure and intermolecular interaction. LWT.

[7] Singhal S., Baker R.D., Baker S.S. (2017). A Comparison of the Nutritional Value of Cow’s Milk and Nondairy Beverages. J. Pediatr. Gastroenterol. Nutr..

[8] Jeske S., Zannini E., Arendt E.K. (2017). Evaluation of Physicochemical and Glycaemic Properties of Commercial Plant-Based Milk Substitutes. Plant Foods Hum. Nutr..

[9] Bernat N., Cháfer M., Chiralt A., González-Martínez C. (2014). Vegetable milks and their fermented derivative products. Int. J. Food Stud..

[10] Codina-Torrella I., Guamis B., Ferragut V., Trujillo A.J. (2017). Potential application of ultra-high pressure homogenization in the physico-chemical stabilization of tiger nuts’ milk beverage. Innov. Food Sci. Emerg. Technol..

[11] Kong D., Li X., Yao J., He Y., Luo J., Yang M. (2020). Health risk assessment and bioaccessibility of toxic elements in edible and medicinal plants under different consumption methods. Microchem. J..

[12] Afonne O.J., Ifediba E.C. (2020). Heavy metals risks in plant foods–need to step up precautionary measures. Curr. Opin. Toxicol..

[13] Islam M.S., Islam A.R.M.T., Phoungthong K., Ustaoğlu F., Tokatli C., Ahmed R., Ibrahim K.A., Idris A.M. (2022). Potentially toxic elements in vegetable and rice species in Bangladesh and their exposure assessment. J. Food Compos. Anal..

[14] Patwa J., Sharma A., Flora S.J.S. (2022). Arsenic, cadmium, and lead. Reproductive and Developmental Toxicology.

[15] Patwa J., Flora S.J.S. (2020). Heavy Metal-Induced Cerebral Small Vessel Disease: Insights into Molecular Mechanisms and Possible Reversal Strategies. Int. J. Mol. Sci..

[16] de Paiva E.L., Medeiros C., Andrekowisk Fioravanti M.I., Milani R.F., Morgano M.A., Lima Pallone J.A., Arisseto-Bragotto A.P. (2020). Aluminium in infant foods: Total content, effect of in vitro digestion on bioaccessible fraction and preliminary exposure assessment. J. Food Compos. Anal..

[17] Aljumaili O.I., El-Waseif A.A., AbdulJabbar Suleiman A., El-Dein A., Ewais E. (2022). Assessment of hair aluminium, cobalt, and mercury in a specimen of autistic Iraqi patients: Environmental risk factors of heavy metals in autism. Mater. Today Proc..

[18] Vidya C.S.-N., Shetty R., Vaculíková M., Vaculík M. (2022). Antimony toxicity in soils and plants, and mechanisms of its alleviation. Environ. Exp. Bot..

[19] Peana M., Medici S., Dadar M., Zoroddu M.A., Pelucelli A., Chasapis C.T., Bjørklund G. (2021). Environmental barium: Potential exposure and health-hazards. Arch. Toxicol..

[20] Tong J., Liang C., Tao S., Geng M., Gan H., Yan S., Cao H., Xie L., Huang K., Tao F. (2023). Association of maternal and cord blood barium exposure with preschoolers’ intellectual function: Evidence from the Ma’anshan Birth Cohort (MABC) study. Sci. Total Environ..

[21] Albin M., Oskarsson A. (2022). Molybdenum. Handbook on the Toxicology of Metals.

[22] FAO (Food and Agriculture Organization of the United Nations), WHO (World Health Organization) (2011). Summary Report of the Seventy-Fourth Meeting of JECFASeventy-Fourth Meeting of the Joint FAO/WHO Expert Committee on Food Additives.

[23] FDA Food and Drug Administration (2013). Guidance for Industry: A Food Labeling Guide—(14. Appendix F: Calculate the Percent Daily Value for the Appropriate Nutrients).

[24] (2013). Brasil Regulamento Técnico MERCOSUL sobre Limites Máximos de Contaminantes Inorgânicos em Alimentos.

[25] (2019). MAPA Consolidação das Normas de Bebidas, Fermentados Acéticos, Vinho e Derivados da Uva e do Vinho. ANEXO À NORMA INTERNA DIPOV N^o^ 01/2019. https://www.gov.br/agricultura/pt-br/assuntos/inspecao/produtos-vegetal/legislacao-1/biblioteca-de-normas-vinhos-e-bebidas/AnexoNormaInternaDIPOVverso301219001.pdf.

[26] (2022). Brasil RESOLUÇÃO DA DIRETORIA COLEGIADA—RDC No 722/22. Dispõe Sobre os Limites máximos Tolerados (LMT) de Contaminantes em Alimentos, os Princípios Gerais para o seu Estabelecimento e os Métodos de Análise para Fins de Avaliação de conformidade.

[27] (2022). Brasil Instrução Normativa—IN N° 160. Limites máximos Tolerados (LMT) de Contaminantes em Alimentos. http://antigo.anvisa.gov.br/documents/10181/2718376/IN_160_2022_.pdf.

[28] INMETRO: The National Institute of Metrology, Stardardization and Industrial Quality (2020). DOQ-CGCRE-008.

[29] AOAC: International, Official Methods of Analysis of AOAC International (2016). Guidelines for Standard Method Performance Requirements (Appendix F).

[30] Fioravanti M.I.A., Milani R.F., de Paiva E.L., Morgano M.A. (2020). Simple and fast ultrasound-assisted method for mineral content and bioaccessibility study in infant formula by ICP OES. Anal. Methods.

[31] Kroes R., Müller D., Lambe J., Löwik M.R., van Klaveren J., Kleiner J., Massey R., Mayer S., Urieta I., Verger P. (2002). Assessment of intake from the diet. Food Chem. Toxicol..

[32] Wang B., Liu Y., Wang H., Cui L., Zhang Z., Guo J., Liu S., Cui W. (2020). Contamination and health risk assessment of lead, arsenic, cadmium, and aluminum from a total diet study of Jilin Province, China. Food Sci. Nutr..

[33] Filippini T., Tancredi S., Malagoli C., Cilloni S., Malavolti M., Violi F., Vescovi L., Bargellini A., Vinceti M. (2019). Aluminum and tin: Food contamination and dietary intake in an Italian population. J. Trace Elem. Med. Biol..

[34] Antoine J.M.R., Fung L.A.H., Grant C.N. (2017). Assessment of the potential health risks associated with the aluminium, arsenic, cadmium and lead content in selected fruits and vegetables grown in Jamaica. Toxicol. Reports.

[35] Codex: Committee on Contaminants in Foods Fifth Session in The Hague. 21–25 March 2011. http://www.fsis.usda.gov/codex/Codex_Committee_Contaminants_in_Foods/index.asp.

[36] Ray A., Jankar J.S. (2022). A Comparative Study of Chromium: Therapeutic Uses and Toxicological Effects on Human Health. J. Pharmacol. Pharmacother..

[37] Anderson R.A. (1997). Chromium as an Essential Nutrient for Humans. Regul. Toxicol. Pharmacol..

[38] Cámara-Martos F., Moreno-Rojas R. (2016). Cobalt: Toxicology. Encyclopedia of Food and Health.

[39] Cubadda F., Iacoponi F., Ferraris F., D’Amato M., Aureli F., Raggi A., Sette S., Turrini A., Mantovani A. (2020). Dietary exposure of the Italian population to nickel: The national Total Diet Study. Food Chem. Toxicol..

[40] Schrenk D., Bignami M., Bodin L., Chipman J.K., del Mazo J., Grasl-Kraupp B., Hogstrand C., Hoogenboom L.R., Nebbia C.S., EFSA CONTAM Panel (EFSA Panel on Contaminants in the Food Chain) (2020). Scientific Opinion on the update of the risk assessment of nickel in food and drinking water. EFSA J..

[41] Arcella D., Cascio C., Gómez Ruiz J., EFSA (European Food Safety Authority) (2021). Scientific report on the chronic dietary exposure to inorganic arsenic. EFSA J..

[42] EFSA CONTAM Panel (EFSA Panel on Contaminants in the Food Chain) (2009). Scientific Opinion on arsenic in food. EFSA J..

[43] JECFA (Joint FAO/WHO Expert Committee on Food Additives) (2011). Safety Evaluation of Certain Contaminants in Food: Prepared by the Seventy-Second Meeting of the Joint FAO/WHO Expert Committee on Food Additives (JECFA).

[44] EFSA NDA Panel (EFSA Panel on Dietetic Products Nutrition and Allergieis) (2013). Scientific Opinion on Dietary Reference Values for molybdenum. EFSA J..

[45] FAO/WHO (Food and Agriculture Organization of the United Nations and World Health Organization) (2011). Evaluation of Certain Food Additives and Contaminants: Seventy-Third [73rd] Report of the Joint FAO/WHO Expert Committee on Food Additives.

[46] EFSA (European Food Safety Authority) (2012). Cadmium dietary exposure in the European population. EFSA J..

[47] US-EPA (United States Environmental Protection Agency) (2001). Integrated Risk Information System. Methylmercury (MeHg).

[48] EFSA Panel on Contaminants in the Food Chain (CONTAM) (2012). Scientific Opinion on the risk for public health related to the presence of mercury and methylmercury in food. EFSA J..

[49] Tylenda C.A., Tomei Torres F.A., Sullivan D.W. (2022). Antimony. Handbook on the Toxicology of Metals.

[50] Oskarsson A. (2022). Barium. Handbook on the Toxicology of Metals.

[51] WHO (World Health Organization) (2004). Barium in Drinking-Water. Background Document for Preparation of WHO Guidelines for Drinking-Water Quality.

[52] Pearson A.J., Ashmore E. (2020). Risk assessment of antimony, barium, beryllium, boron, bromine, lithium, nickel, strontium, thallium and uranium concentrations in the New Zealand diet. Food Addit. Contam. Part A.

[53] Mielech A., Puścion-Jakubik A., Socha K. (2021). Assessment of the Risk of Contamination of Food for Infants and Toddlers. Nutrients.

[54] EFSA Panel on Contaminants in the Food Chain (CONTAM) (2010). Scientific Opinion on Lead in Food. EFSA J..

